# CXCR2–CXCL1 axis is correlated with neutrophil infiltration and predicts a poor prognosis in hepatocellular carcinoma

**DOI:** 10.1186/s13046-015-0247-1

**Published:** 2015-10-26

**Authors:** Li Li, Li Xu, Jing Yan, Zuo-Jun Zhen, Yong Ji, Chao-Qun Liu, Wan Yee Lau, Limin Zheng, Jing Xu

**Affiliations:** Collaborative Innovation Center for Cancer Medicine, Sun Yat-sen University Cancer Center, Guangzhou, P. R. China; Department of Hepatic & Pancreatic Surgery, The First People’s Hospital of Foshan, Foshan, Guangdong P. R. China; Faculty of Medicine, The Chinese University of Hong Kong, Shatin, New Territories, Hong Kong, SAR P. R. China; Department of Medicine, Division of Rheumatology, Immunology and Allergy, Brigham and Women’s Hospital, One Jimmy Fund Way, Boston, MA 02115 USA

**Keywords:** Hepatocellular carcinoma, Chemokine, CXCR2, CXCL1, Immune cell infiltration, Prognosis

## Abstract

**Background & aims:**

Inflammation is a hallmark of cancer, yet the mechanisms that regulate immune cell infiltration into tumors remain poorly characterized. This study attempted to characterize the composition, distribution, and prognostic value of CXCR2^+^ cells in hepatocellular carcinoma (HCC) and to examine the CXCR2 ligands that are responsible for local immune infiltration in different areas of HCC tumors.

**Methods:**

Immunohistochemistry and immunofluorescene were used to identify CXCR2^+^ cells in HCC tissues. Kaplan–Meier analysis and Cox regression models were applied to estimate recurrence-free survival (RFS) and overall survival (OS) for 259 HCC patients. The expression levels of CXCR2 ligands (CXCL-1, −2, −5, and −8) were measured by real-time PCR and compared with local immune cell density. The combined prognostic value of the CXCR2–CXCL1 axis was further evaluated.

**Results:**

In HCC tissues, CXCR2^+^ cells were mainly neutrophils that were enriched in the peri-tumoral stroma (PS) region. Kaplan–Meier survival analysis showed that increased CXCR2^+^_*PS*_ cells were associated with reduced RFS and OS (*P* = 0.015 for RFS; *P* = 0.002 for OS). Multivariate Cox proportional hazards analysis identified CXCR2^+^_*PS*_ cell density as an independent prognostic factor for OS (hazard ratio [HR] = 1.737, 95 % confidence interval [CI] = 1.167–2.585, *P* = 0.006). Furthermore, we detected a positive correlation between the density of CD15^+^ neutrophils and CXCL1 levels in both the peri-tumoral stroma and intra-tumoral regions. The combination of CXCR2 and CXCL1 expression levels represented a powerful predictor of a poor prognosis for patients with HCC.

**Conclusions:**

Our data showed that the CXCR2^+^ cell density was an independent prognostic factor for predicting OS for HCC patients. The CXCR2–CXCL1 axis can regulate neutrophil infiltration into HCC tumor tissues and might represent a useful target for anti-HCC therapies.

**Electronic supplementary material:**

The online version of this article (doi:10.1186/s13046-015-0247-1) contains supplementary material, which is available to authorized users.

## Background

Hepatocellular carcinoma (HCC) is an aggressive malignancy with an increasing rate of incidence and a poor prognosis [1]. Surgical intervention represents the most effective therapy for HCC; however, more than 70 % of patients relapse within 5 years, and overall survival remains poor [2]. Although several types of adjuvant therapy have been reported to reduce the recurrence rate, the benefits of these approaches are limited [3]. Thus, an urgent unmet need exists to gain a better understanding of the underlying mechanisms that lead to tumor progression and to identify prognostic biomarkers for HCC patients.

HCCs are typically attributed to chronic hepatitis B or C viral infections and, thus, present with extensive immune cell infiltration [4, 5]. A growing number of studies indicate that immune cells in different tumor milieus can exhibit diverse functions in that regulate tumor growth, invasion, and angiogenesis [6–9]. Our previous studies have shown that activated monocytes in peri-tumoral regions can attenuate T cell responses by expressing PD-L1, whereas suppressive macrophages in a tumor nest can induce the generation of regulatory T (Treg) cells to promote tumor progression [10–12]. We also found that neutrophils accumulated in the peri-tumoral regions of HCC where they promoted angiogenesis by releasing MMP9 [13]. Accordingly, the density of macrophages and neutrophils were both correlated with patient prognoses in HCC. However, it remains unclear how these diverse types of immune cells can be selectivity recruited to different tumor sites.

Chemokines are low molecular weight (8 to 14 kDa) chemotactic cytokines with diverse bioactivities that can mediate the recruitment and trafficking of leukocytes to tumor microenvironments [14]. Different tumors exhibit distinct chemokine expression profiles, which can lead to diverse immune cell infiltration and clinical outcomes. CXC receptor 2 (CXCR2) is a member of the G-protein-coupled receptor superfamily. Previous studies have revealed that CXCR2 and its ligands (CXCL-1, −2, −3, −5, −6, −7, and −8) are involved in tumor angiogenesis, tumorigenesis, and metastasis of several types of human cancers, such as colorectal carcinoma, melanoma, lung cancer, renal cell carcinoma, prostate carcinoma, pancreatic carcinoma, and esophageal carcinoma [15–21]. CXCR2 expression has been detected on monocytes, macrophages, lymphocytes, neutrophils, mast cells, and endothelial cells [14, 22]. However, the composition and distribution of CXCR2^+^ cells, and the role of these cells in immune modulation in HCC has been not been well characterized.

Herein, we investigate the expression and prognostic significance of CXCR2 expression in HCC patients. We also explore the possible involvement of CXCR2 and its ligands in regulating immune cell infiltration into HCC tissues. Our findings show that the CXCR2–CXCL1 axis is correlated with CD15^+^ neutrophil infiltration into both intra- and peri-tumoral regions, and represents an independent prognostic factor in HCC.

## Materials and methods

### Patients and specimens

Archived, formalin-fixed, paraffin-embedded tissues from 259 patients (Group 1) who had all undergone curative resection for HCC at the Sun Yat-Sen University Cancer Center between 2007 and 2010 were enrolled in this prognostic study. Patients who exhibited signs of distant metastasis, had received anti-cancer therapies prior to surgery, or experienced concurrent autoimmune disease were excluded. The diagnosis of HCC in each patient was confirmed histopathologically. Curative resection for HCC was defined as complete resection of all tumor nodules with a resection margin of at least 1 cm, and with a cut surface that was free of tumors based on histological examinations. To ensure the complete removal of HCC, intraoperative ultrasound and postsurgical contrast-enhanced computed tomography (CT) were routinely used [13, 23, 24]. The tumor stage was determined according to the Barcelona-Clinic Liver Cancer (BCLC) staging classification and tumor-node-metastasis (TNM) classification system of the International Union Against Cancer, 7th Edition [25]. Tumor differentiation was graded according to the Edmondson grading system. Liver function was assessed based on the Child–Pugh scoring system. Data was censored at the last follow-up for patients without recurrence or death. Recurrence-free survival (RFS) was defined as the time from the date of surgery to either recurrence, the last follow-up, or death if no recurrence was observed. Overall survival (OS) was defined as the interval between the time of surgery to either the last follow-up or death.

Frozen and matched formalin-fixed, paraffin-embedded tissues from 30 HCC patients (Group 2) who received therapy between 2012 and 2013 were used for real-time PCR and tissue microarray (TMA) analyses, respectively.

This study strictly conformed to the ethical guidelines of the Declaration of Helsinki and was approved by the Research Ethics Committee of Sun Yat-Sen University Cancer Center. Written informed consent was obtained from all patients prior to sample collection. All samples were coded and data was stored anonymously. The clinicopathological characteristics of all patients are summarized in Table 1.

**Table 1:** Clinical characteristics of patients with HCC

Variables	Group 1	Group 2
Cases (n)	259	30
Age, years (median, range)	52, 13–79	42,26–80
Gender (male / female)	226/33	29/1
HBsAg (negative / positive)	20/239	1/29
Cirrhosis (absent / present)	96/163	6/24
ALT (U/liter, ≤42 / >42)	154/105	16/14
AST (U/liter, ≤42 / >42)	150/109	18/12
AFP (ng/ml, ≤25 / >25)	89/170	11/19
Tumor size (cm, ≤5 / >5)	109/150	12/18
Tumor differentiation (I–II / III–IV)	133/124	16/14
Vascular invasion (absent / present)	210/49	18/12
Tumor multiplicity (solitary / multiple)	194/65	17/13
TNM stage (I–II / III–IV)	139/120	22/8
BCLC stage (0–A vs. B–C)	152/107	10/20

*Abbreviations*: *AFP* alpha-fetoprotein, *ALT* alanine aminotransferase, *AST* aspartate aminotransferase, *BCLC* Barcelona Clinic Liver Cancer, *HBsAg* hepatitis B surface antigen, *TMA* tissue microarray, *TNM* tumor-nodes-metastasis

### Tissue microarray and immunohistochemistry

Immunohistochemistry (IHC) for detecting CXCR2 and CXCL1 was carried out on paraffin-embedded sections from 259 patients. IHC for CD3, CD15, S100, and CD68 was performed on TMA sections that contained 30 specimens.

The TMA was constructed as described previously [23, 24]. Briefly, each representative area was premarked in the paraffin-embedded blocks by hematoxylin and eosin (H&E) staining, excluding necrotic and hemorrhagic areas. To ensure reproducibility and homogeneity, duplicates of 2 mm (diameter) cylinders from the peri-tumoral stroma region, and 1 mm (diameter) cylinders from the intra-tumoral and non-tumor regions were obtained from each patient.

IHC was performed using a two-step protocol (DakoCytomation, Glostrup, Denmark) using protocols described in our previous studies [23, 24]. Sections of formalin-fixed, paraffin-embedded tissues were cut using a microtome, and then were sequentially dried, dewaxed, and re-hydrated with xylene and a decreasing ethanol series. Next, endogenous peroxidase activity was blocked with 0.3 % H_2_O_2_ for 10 min. For antigen retrieval, sections were steamed in 10 mM citrate buffer (pH 6.0) for 10 min. Glass slides were incubated overnight at 4 °C with anti-CXCR2 (Monoclonal Mouse IgG2A; Clone 48311; R&D Systems, Minneapolis, MN, USA), anti-CXCL1 (Polyclonal Rabbit Antibody, LifeSpan BioSiences, Seattle, WA, USA), anti-CD3 (Monoclonal Rabbit IgG; Clone SP7, Thermo Scientific, Waltham, MA, USA), anti-CD15 (Monoclonal Mouse IgM; Clone LeuM1; Beijing Zhongshan Golden Bridge Biotechnology, Beijing, China), anti-S100 (Monoclonal Mouse IgG2A; Clone 4C4.9; Thermo Scientific), or anti-CD68 (Monoclonal Mouse IgG3; Clone PG-M1; DakoCytomation, Carpinteria, CA, USA) antibodies. Horseradish peroxidase-conjugated anti-rabbit and anti-mouse Dako EnVision systems (DakoCytomation) were used as secondary detection reagents that were developed using 3,3′-diaminobenzidine (DAB). All sections were lightly counterstained with Mayer’s Hematoxylin Solution (Sigma) and mounted using non-aqueous Permount TM mounting medium. Negative controls were slides for which the primary antibodies were replaced by the same concentration of an irrelevant, isotype-matched antibody. Brown precipitate indicated positive immunoreactivity.

### Immunofluorescent staining

For double-immunofluorescence of CD3, CD15, CD68, or S100 along with CXCR2, a fluorescein tyramide signal amplification (TSA) system (TSA-Plus Fluorescence Palette System, PerkinElmer) was used according to the manufacturer’s instructions. Fluorescent signals were produced using Cy3, fluorescein, or Cy5 TSA systems. Nuclei were counterstained using DAPI.

### Automated image acquisition and quantification

To quantify CXCR2 expression, the Vectra-Inform image analysis system (Perkin-Elmer/Applied Biosystems, Foster City, CA, USA) was used as described in previous studies [24]. Briefly, slides were loaded onto a Vectra slide scanner following the manufacturer’s protocol. Representative areas for tissues from each patient were collected in 10–12 fields from each slide at a constant 200× magnification. Stained slides were imaged using a Nuance VIS-FL Multispectral Imaging System (Perkin-Elmer/Applied Biosystems). The spectrum for each chromogen was determined on single-stained control slides. A spectral un-mixing algorithm separated grayscale images that quantitatively captured each spectral component. Images were analyzed with InForm 2.0.1 image analysis software (Perkin-Elmer Applied Biosystems).

For staining with DAB and hematoxylin, a standard bright field scanning protocol was developed. Using InForm software, the tissue (blank, tumor tissue, peri-tumoral stroma tissue) and cell (nucleus, cytoplasm) compartments could be segregated [24]. Target signals were quantified in selected tissues and cellular compartments of interest. For a given area of tissue, the category area (percentage), number of cells, positivity, and DAB object density counts per megapixel were used in subsequent analyses. The percentage of each immune cell subset (CD3, CD15, and CD68) was calculated by dividing the absolute number of each cell subset by the total number of infiltrating immune cells.

To quantify immunofluorescence, a standard fluorescence-field scanning protocol was developed. Proportions of cells in different populations were calculated [e.g., the proportion of CXCR2^+^ cells that were CD15^+^ neutrophils would be calculated as: (number of CXCR2^+^CD15^+^ cells) / (number of CD15^+^CXCR2^+^ cells + CD15^−^CXCR2^+^ cells].

### Real-time quantitative RT-PCR and gene expression analysis

Total RNA was extracted from HCC tumor samples and corresponding peri-tumoral and non-tumoral liver tissues using TRIzol reagent (Invitrogen) according to the manufacturer’s instructions. Equal concentrations of total RNA were used in reverse transcription reactions to generate cDNA using a miScript Reverse Transcription Kit (Qiagen, Hilden, Gemany), then each cDNA was used in SYBR Green real-time quantitative PCR according to standard protocols; primer sequences are listed in Additional file 1: Table S1. All real-time quantitative PCR was performed on a Roche LightCycler480 (Roche Diagnostics). Data were generated using LightCycler480 software (version 1.5.0). Levels of target genes were normalized to levels of the expression of a reference gene, *GAPDH*. Data were processed using GraphPad Prism software (La Jolla, CA, USA).

### Statistical analyses

RFS and OS curves were obtained using the Kaplan–Meier method, and compared using the log-rank test for each prognostic variable. Variables with effects on survival in univariate analysis were included in a multivariate Cox proportional hazard regression model, which was used to estimate the adjusted hazard ratio (HR) and 95 % confidence interval (CI) and to identify independent prognostic factors. Subgroups of each immunostaining parameter were divided by median values. Associations between immunostaining parameters and clinicopathological features were evaluated using the *χ*^2^ test or Fisher’s exact test as appropriate. Associations between numbers of tumor-infiltrating immune cells and the expression levels of CXCR2 ligands were calculated using Pearson’s test. A threshold of *P* <0.05 was set to denote statistical significance. SPSS 17.0 (IBM) was used for statistical analyses.

## Results

### CXCR2 expression in HCC tissues

**Fig. 1:**
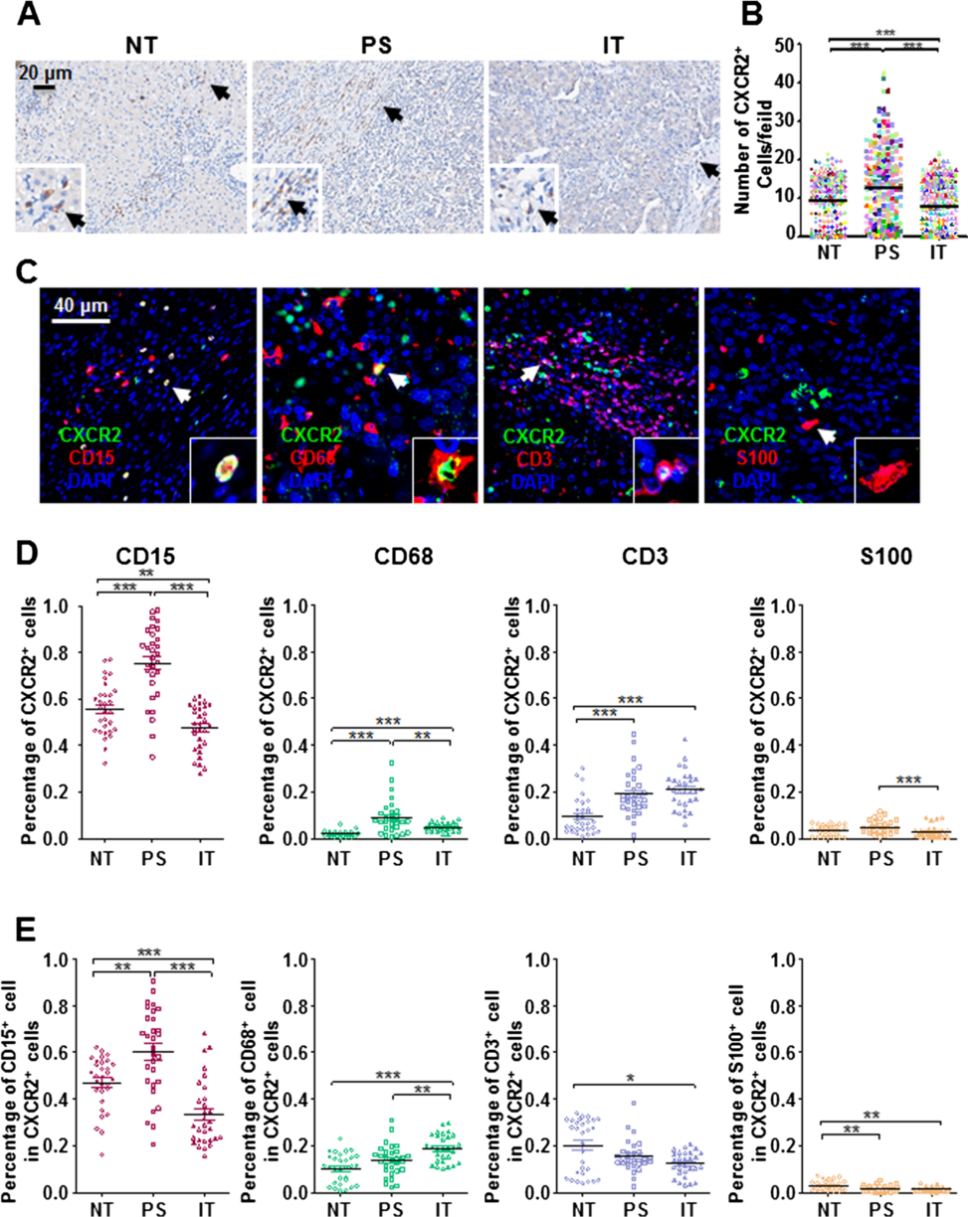
*In situ* CXCR2 expression in HCC tumors. **a** Representative images show CXCR2^+^ cells stained brown in non-tumor (NT), peri-tumoral stroma (PS), and intra-tumor (IT) regions in HCC tissues. Black arrows indicate CXCR2^+^ cells. **b** The densities of CXCR2^+^ cells in the NT, PS, and IT regions of HCC tissues were calculated (*n* = 259). Results are expressed as means ± SEM (bars) of each group. **c** Multiple stains for CD15, CD68, CD3, or S100 (*red*), CXCR2 (*green*), and DAPI (*blue*) in paraffin-embedded sections were developed using a TSA system. White arrows indicate representative cells that express CXCR2. **d** Vectra-Inform image analysis of the proportion of CXCR2^+^ cells among CD15^+^, CD68^+^, CD3^+^, or S100^+^ cells in HCC tissues (*n* = 30). Results are expressed as means ± SEM (bars). **e** Proportions of CD15^+^, CD68^+^, CD3^+^, or S100^+^ cells among CXCR2^+^ cells in HCC tissues (*n* = 30). Results are expressed as means ± SEM (bars); *, *P* <0.05; **, *P* <0.01; ***, *P* <0.001

To assess CXCR2 expression in HCC tumors, we performed IHC staining in paraffin-embedded sections. CXCR2 signals were mainly identified in the cytoplasm of stromal cells (Fig. 1a). CXCR2^+^ cells were dispersed and found to be enriched in peri-tumoral stroma (PS) compared with non-tumoral (NT) or intra-tumoral (IT) regions, with mean (± SEM) densities of 12.61 ± 0.61, 9.39 ± 0.39, and 7.98 ± 0.40, respectively (Fig. 1b).

Multiple immunofluorescence staining for CXCR2 and various immune cell markers (CD15, neutrophils; CD68, macrophages; CD3, T cells; S100, dendritic cells) was performed (Fig. 1c), and then images were analyzed using the Vectra-Inform image analysis system to calculate the proportion of CXCR2^+^ cells within each cellular subset in HCC tissues. As shown in Fig. 1, most CXCR2^+^ cells were CD15^+^ neutrophils in the NT, PS, and IT regions of HCC tissues (49.6 % ± 2.1 %, 64.0 % ± 3.4 %, and 28.7 % ± 2.6 %, respectively; *n* = 30; Fig. 1e), whereas only a small portion of CXCR2^+^ cells were CD68^+^ macrophages (11.1 % ± 1.1 %, 14.4 % ± 1.2 %, and 18.7 % ± 1.0 %, respectively), CD3^+^ T cells (26.1 % ± 2.1 %, 14.1 % ± 1.2 %, and 13.2 % ± 0.9 %), or S100^+^ dendritic cells (2.4 % ± 0.4 %, 1.2 % ± 0.2 %, and 1.3 % ± 0.2 %). Moreover, the proportion of CD15^+^ neutrophils that expressed CXCR2 in the NT, PS, and IT regions was 55.2 % ± 2.0 %, 78.6 % ± 2.9 %, and 48.9 % ± 1.8 %, respectively (Fig. 1d).

### Increased CXCR2^+^ cells predicted poor survival

**Fig. 2:**
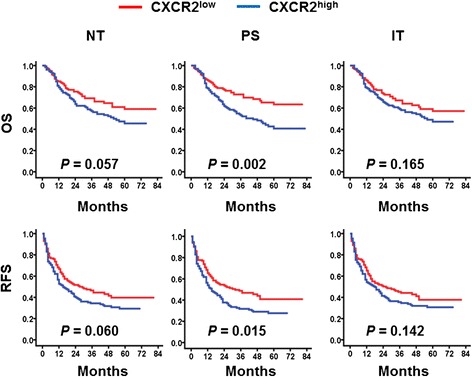
Accumulation of CXCR2^+^ cells predicts poor survival in HCC. OS (*top*) and RFS (*bottom*) were estimated using the Kaplan–Meier method and compared using the log-rank test (*n* = 259)

The relationship between CXCR2^+^ cells and patient survival was further investigated. A cohort of 259 HCC patients who had received curative resection were divided into two groups based on the median value of CXCR2^+^ cell density in NT (CXCR2^+^_*NT*_ cells; median density, 10.87), PS (CXCR2^+^_*PS*_ cells; median density, 12.45) and IT (CXCR2^+^_*IT*_ cells; median density, 8.22) areas. Kaplan–Meier survival analysis revealed a negative association between the density of CXCR2^+^_*PS*_ cells and the RFS and OS of patients (*P* = 0.015 for RFS, *P* = 0.002 for OS; log-rank test; Fig. 2). An increased density of CXCR2^+^_*PS*_ cells was associated with a shorter RFS (median, 15 months) and OS (median, 45 months) compared with patients exhibiting a low CXCR2^+^_*PS*_ cell density (median, 32 months and >72 months, respectively). In patients with a high density of CXCR2^+^_*PS*_ cells, the 5-year RFS and OS rates were only 27.7 and 40.7 %, compared with 40.8 and 63.4 % in patients with a low CXCR2^+^_*PS*_ cell density, respectively. However, no association was observed for CXCR2^+^ cells in the NT or IT regions (Fig. 2).

To assess whether CXCR2 could be used as an independent predictor of survival, we performed multivariate Cox proportional hazards analysis. CXCR2^+^_*PS*_ cell density was associated with an elevated risk of recurrence (HR = 1.358, 95 % CI = 0.989–1.866, *P* = 0.059) and death (HR = 1.737, 95 % CI = 1.167–2.585, *P* = 0.006). Clinicopathological variables that were shown to be significant in the univariate analysis were used as covariates in the multivariate analysis, and the CXCR2^+^_*PS*_ cell density was found to be an independent prognostic factor for OS (Table 2). In addition to CXCR2, the multivariate Cox proportional hazards analysis showed that serum AFP levels also represented an independent predictor of OS (*P* = 0.029).

**Table 2:** Univariate and multivariate analyses of factors associated with survival and recurrence

	OS				RFS			
	Univariate	Multivariate			Univariate	Multivariate		
Variables	*P*-value	HR	95 % CI	*P*-value	*P*-value	HR	95 % CI	*P*-value
Age (>52 vs. ≤52 years)	0.438			NA	0.278			NA
Gender (male vs. female)	0.768			NA	0.407			NA
HBsAg (positive vs. negative)	0.372			NA	0.065			NA
Cirrhosis (present vs. absent)	0.278			NA	0.253			NA
ALT (>42 vs. ≤42 U/L)	0.206			NA	0.779			NA
AST (>42 vs. ≤42 U/L)	*0.001*	1.416	0.948–2.116	0.089	*0.013*	1.228	0.883–1.709	0.222
AFP (>25 vs. ≤25 ng/ml)	*0.001*	1.680	1.055–2.675	*0.029*	*0.005*	1.356	0.953–1.930	0.090
Tumor size (>5 vs. ≤5 cm)	*0.002*	1.134	0.702–1.832	0.609	*0.016*	1.018	0.694–1.493	0.929
Tumor differentiation (III–IV vs. I–II)	0.153			NA	0.377			NA
Vascular invasion (present vs. absent)	*<0.001*	1.698	0.889–3.242	0.109	*0.010*	1.341	0.773–2.328	0.297
Tumor multiplicity (multiple vs. solitary)	*<0.001*	1.276	0.664–2.452	0.464	*<0.001*	1.452	0.845–2.495	0.177
TNM stage (III–IV vs. I–II)	*<0.001*	1.301	0.752–2.250	0.346	*<0.001*	1.300	0.835–2.024	0.246
BCLC stage (B–C vs. 0–A)	*<0.001*	1.235	0.559–2.730	0.602	*<0.001*	1.061	0.558–2.020	0.856
CXCR2^+^_*NT*_ cell (high vs. low)	0.060			NA	0.066			NA
CXCR2^+^_*PS*_ cell (high vs. low)	*0.002*	1.737	1.167–2.585	*0.006*	*0.018*	1.358	0.989–1.866	0.059
CXCR2^+^_*IT*_ cell (high vs. low)	0.169			NA	0.151			NA

Note: The Cox proportional hazards regression model was used. Variables used in multivariate analysis were adopted by univariate analysis. Terms in italics indicate statistical significance

*Abbreviations*: *AFP* alpha-fetoprotein, *ALT* alanine aminotransferase, *AST* aspartate aminotransferase, *BCLC* Barcelona Clinic Liver Cancer, *CI* confidence interval, *HBsAg* hepatitis B surface antigen, *HR* hazard ratio, *NA* not adopted, *TNM* tumor-nodes-metastasis

We also tested whether there were any significant associations between the CXCR2 density and clinicopathological variables. The density of CXCR2^+^ cells was significantly increased in HCC patients with less-differentiated NT, PS, and IT regions (*P* = 0.015, *P* = 0.040, and *P* = 0.040, respectively; Additional file 2: Table S2).

### Associations between CXCR2 ligand expression and local immune cell infiltration

**Fig. 3:**
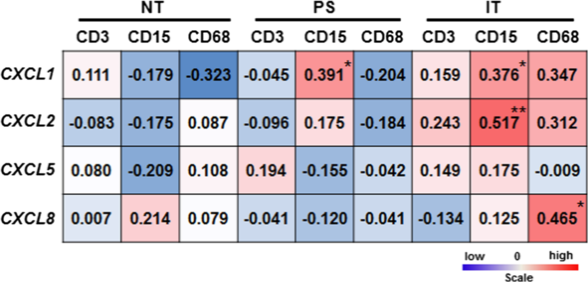
Correlations between *CXCL-1*, −*2*, −*5*, and −*8* expression and CD3^+^, CD15^+^, and CD68^+^ cell densities in HCC tissues. Correlation coefficients between *CXCL-1*, −*2*, −*5*, and −*8* expression and densities of each immune cell subset are shown; *, *P* <0.05; **, *P* <0.01

To further assess the expression of CXCR2 ligands in HCC, we analyzed the mRNA transcript levels of *CXCL-1*, −*2*, −*5*, and −*8* in 30 HCC tumor samples with matched peri-tumoral and non-tumorous liver tissues (Additional file 3: Figure S1), We also examined local immune cell infiltration in the same samples by IHC staining for CD3, CD15, and CD68. Consistent with our previous findings, CD15^+^ cells and CD3^+^ T cells (Additional file 4: Figure S2) were predominantly enriched in the PS region surrounding the tumor nest at the tumor edge [13, 23]. Correlations between *CXCL-1*, −*2*, −*5*, and −*8* expression and the density of CD3^+^, CD15^+^, and CD68^+^ cells in HCC tissues were examined using Pearson’s correlation test. We detected a positive correlation between the density of CD68^+^ cells and *CXCL8* expression (R = 0.465; *P* = 0.011; Fig. 3) and a positive correlation between the density of CD15^+^ cells and *CXCL1* and *CXCL2* expression (R = 0.376, *P* = 0.040; and R = 0.517, *P* = 0.003, respectively; Fig. 3) in the IT region. However, in the PS region, the density of CD15^+^ cells was only correlated with *CXCL1* expression (R = 0.391, *P* = 0.033; Fig. 3). These findings indicated that the CXCL1–CXCR2 axis may play an important role in promoting CD15^+^ neutrophil accumulation in HCC tumors.

### Combination of CXCL1 and CXCR2 exhibits improved prognostic power for HCC

Our aforementioned findings indicated that the density of CXCR2^+^ cells represented a valuable independent factor for predicting the prognosis of HCC, and CXCL1 was a key ligand responsible for mediating neutrophil infiltration into HCC tumor tissues. Therefore, we analyzed whether the combination of CXCR2 and CXCL1 represented a more powerful criteria for predicting patient prognoses.

**Fig. 4:**
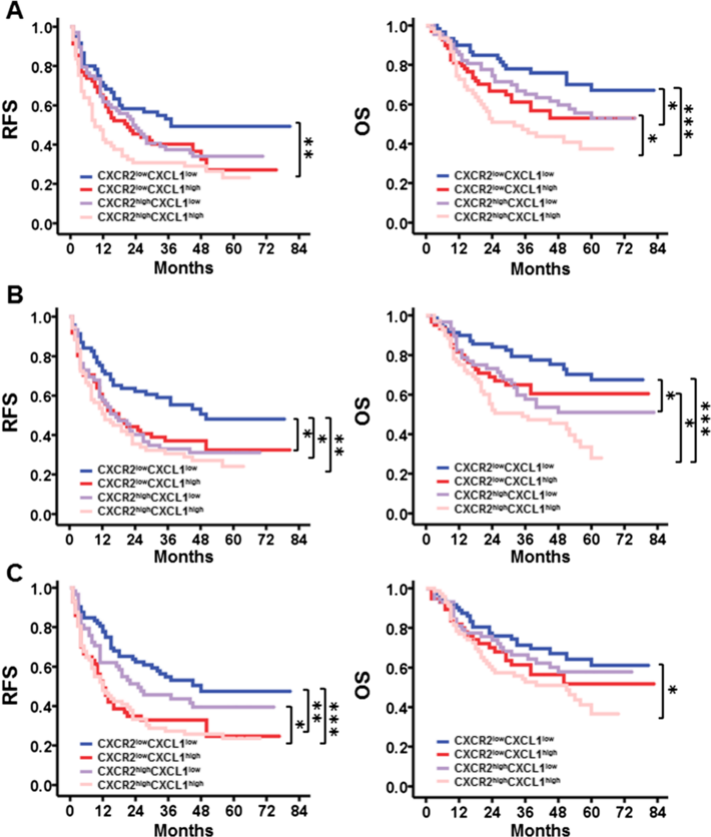
Prognostic significance of the combined actions of CXCR2 and CXCL1 expression in association with RFS and OS. Kaplan–Meier curves illustrate the duration of RFS and OS according to the combination of CXCR2 and CXCL1 expression in non-tumoral (**a**), peri-tumoral stroma (**b**) and intra-tumoral (**c**) regions; *, *P* <0.05; **, *P* <0.01; ***, *P* <0.001

To determine the relationship between CXCL1 expression and patient survival, CXCL1 expression was also divided by the median CXCL1 object density per megapixel (median NT, PS and IT CXCL1: 292.2, 337.8, and 418.6, respectively). In all three regions, patients in the CXCR2^low^CXCL1^low^ group exhibited a better RFS (median RFS: 37 months for NT, 50 months for PS, and 48 months for IT) and OS (median OS > 72 months for NT, PS, and IT) than those in the CXCR2^high^CXCL1^high^ group (median RFS: 9 months for NT, 13 months for PS, and 12 months for IT; median OS: 32 months for NT, 35 months for PS, and 51 months for IT; Fig. 4). In the multivariate Cox analysis, a combination of CXCR2 and CXCL1 in the PS region was associated with an elevated risk of death (HR = 1.194, 95 % CI = 1.044–1.366; *P* = 0.010; Additional file 5: Table S3). These findings suggested that CXCR2 and CXCL1 represented a powerful predictor of shorter OS for patients with HCC.

## Discussion

In this present study, we found that most CXCR2^+^ cells in HCC tumors were neutrophils, and that CXCR2 represents an independent prognostic factor for HCC after resection. Patients with fewer CXCR2^+^ cells within the peri-tumoral stroma had a significantly prolonged RFS and OS compared with those patients with more CXCR2^+^ cells. Multivariate analysis showed that CXCR2 expression in the peri-tumoral stroma could also serve as a useful biomarker for predicting the prognosis of HCC. Furthermore, by analyzing associations between CXCR2 ligand expression and immune cell infiltration, we found a significant correlation between CXCL1 expression and CD15^+^ neutrophil infiltration in both the PS and IT regions. A combination of CXCR2 and CXCL1 expression could improve the prognostic power for predicting survival. These findings suggest the possible involvement of the CXCR2–CXCL1 axis in regulating neutrophil infiltration to promote HCC.

The chemokine network represents a unique group of cytokines and receptors that exhibit various bioactivities that can mediate the recruitment and trafficking of leukocytes to tumor microenvironments. Various immune cells can be recruited into tumors by diverse chemokines [26]. Neutrophils represent an important component of the intra-tumoral leukocyte infiltrate. Recent studies have reported that both CXCR6 and CXCL5 are correlated with intra-tumoral neutrophil infiltration in HCC [27, 28]. In this present study, we showed that compared to HCC tissue and tumor-adjacent normal tissues, the peri-tumoral stroma exhibited the highest density of infiltrating CXCR2^+^ cells, which were mainly CD15^+^ neutrophils. By quantitative RT-PCR, we observed positive correlation of peri- and intra-tumoral CXCL1 expression with the density of CD15^+^ neutrophils. It has been reported that CXCL1 can mediate neutrophil migration in models of mouse lung inflammation and experimental arthritis [29]. Together, our results indicate that the CXCR2–CXCL1 axis is likely responsible for regulating neutrophil inflation in HCC tumors, which might further influence patient prognoses by facilitating tumor angiogenesis [13].

Chemokines and chemokine receptors can affect multiple pathways that contribute to tumor progression. Previous studies have reported roles for CXCR2 in tumor development [30]. However, the functional roles of CXCR2 in tumor cells remain the subject of debate. CXCR2 has been shown to promote cell proliferation, invasion, and migration, while CXCR2 inhibitors have been reported to reduce tumor growth [31]. By contrast, a recent study reported the induction of CXCR2 and CXCR2 ligand expression by oncogenic K-ras, which reinforces senescence *in vitro* and suggests that it acts as a tumor suppressor [32]. Chemokines can exert direct effects on tumor cells and recruit various cell types to the tumor microenvironment, which can further affect the immune status of a tumor. Herein, we examined the expression of CXCR2 in HCC tissues, and found the CXCR2 could indeed be expressed by different types of immune cells, mainly neutrophils. The density of CXCR2^+^ cells was inversely correlated with patient prognoses. Therefore, we confirmed that CXCR2 expression on immune cells likely plays a vital role in tumor progression.

The common ligands for CXCR2 are CXCL1, CXCL2, CXCL5, and CXCL8. CXCL1 and CXCL2 are the main ligands for CXCR2, which can mediate its roles in metastasis and chemoresistance. Recent studies have shown that CXCL1/2 can be hyperactivated in breast cancer cell lines by chemotherapy and that blocking CXCR2 in conjunction with chemotherapeutic agents could markedly reduce lung metastases in xenograft-implanted mice [33]. CXCL5 has been reported to be up-regulated in malignant tumors, such as nasopharyngeal and colorectal cancer, and to be closely correlated with a poor prognosis [27, 34–36]. In HCC, Zhou et al. demonstrated that the CXCR2–CXCL5 axis can contribute to the EMT and HCC metastasis through activation of PI3K/Akt/GSK-3β/Snail signaling [37]. CXCL8 expression has been documented in infiltrating neutrophils, tumor-associated macrophages, tumor cells, and endothelial cells, and has been shown to regulate tumor angiogenesis, tumor cell proliferation, and metastasis potential, such as vessel invasion in HCC [38]. In this present study, we found that CXCL8 was positively correlated with CD68 expression in the IT region, which indicated that CXCL8 might be responsible for macrophage recruitment into tumor nests, which adds to the complexity of CXCR2-mediated leukocyte recruitment to the HCC tumor microenvironment.

## Conclusions

Our study showed that most CXCR2^+^ cells are CD15^+^ neutrophils in HCC tissues. The CXCR2–CXCL1 axis may be responsible for regulating neutrophil infiltration in HCC and might represent a potential therapeutic target for treating HCC.

## Additional files

Additional file 1: Table S1. Primer Sequences. (DOC 58 kb) Additional file 2: Table S2. Association of CXCR2 with clinicopathological characteristics. (DOC 66 kb) Additional file 3: Figure S1. The gene expression of *CXCL1, CXCL2, CXLC5* and *CXCL8* in different regions of HCC tissues. Total RNA was extracted from HCC tumor samples and corresponding peri-tumoral and non-tumoral liver tissues. Equal concentrations of total RNA were used in reverse transcription reactions to generate cDNA, then each cDNA was used in SYBR Green real-time quantitative PCR. Data were generated using LightCycler480 software. Levels of target genes were normalized to levels of the expression of a reference gene, *GAPDH*. Data were processed using GraphPad Prism software; **, *P* <0.01; ***, *P* <0.001. (TIFF 45 kb) Additional file 4: Figure S2. The number of CD15^+^, CD68^+^, CD3^+^, and S100^+^ cells in different regions of HCC tissues. Paraffin-embedded HCC sections were stained with anti-CD15, CD68, CD3, or S100 antibodies. The density of CD15^+^, CD68^+^, CD3^+^, and S100^+^ cells in non-tumoral (NT), peri-tumoral stroma (PS), and intra-tumoral (IT) regions of the same tissue block were calculated (*n* = 30). Results are expressed as means ± SEM (bars) of groups; *, *P* <0.05; ***, *P* <0.001. (TIFF 44 kb) Additional file 5: Table S3. Univariate and multivariate analyses of factors associated with survival and recurrence. (DOC 40 kb)

## Abbreviations

HCC: Hepatocellular carcinoma
CXCR2: CXC receptor 2
CXCL1: CXC ligand 1
RFS: Recurrence-free survival
OS: Overall survival
PD-L1: Programmed cell death 1 ligand 1
Treg: Regulatory T
MMP9: Matrix metalloproteinase 9
BCLC: Barcelona-Clinic Liver Cancer
TNM: Tumor-node-metastasis
IHC: Immunohistochemistry
DAB: 3,3′-diaminobenzidine
TMA: Tissue microarray
TSA: Tyramide signal amplification
HR: Hazard ratio
CI: Confidence interval
PS: Peri-tumoral stroma
NT: Non-tumoral
IT: Intra-tumoral
AFP: Alpha-fetoprotein
ALT: Alanine aminotransferase
AST: Aspartate aminotransferase
HBsAg: Hepatitis B surface antigen
NA: Not adopted
