# Supplementary material for: CXCR2–CXCL1 axis is correlated with neutrophil infiltration and predicts a poor prognosis in hepatocellular carcinoma
Source: J Exp Clin Cancer Res. 2015 Oct 26;34:129. doi: 10.1186/s13046-015-0247-1 (PMC4621872; doi:10.1186/s13046-015-0247-1)
Supplement: Additional file 1: Table S1. — Primer Sequences. (DOC 58 kb) [file 13046_2015_247_MOESM1_ESM.doc]

**Additional file 1: Table S1.** Primer Sequences.

| *CXCL1*-F | 5’-CAAACCGAAGTCATAGCCACAA-3’ |
| --- | --- |
| *CXCL1*-R | 5’-CTCCTAAGCGATGCTCAAACA-3’ |
| *CXCL2*-F | 5’-CCCAAACCGAAGTCATAGCC-3’ |
| *CXCL2*-R | 5’-CAGGAACAGCCACCAATAAGC-3’ |
| *CXCL5*-F | 5’-AGACCACGCAAGGAGTTCATC-3’ |
| *CXCL5*-R | 5’-GTTCTTCAGGGAGGCTACCAC-3’ |
| *CXCL8*-F | 5’-TGAATGGGTTTGCTAGAATGTG-3’ |
| *CXCL8*-R | 5’-ACTGTGAGGTAAGATGGTGGCT-3’ |
| *GAPDH*-F | 5’-CCAGCAAGAGCACAAGA-3’ |
| *GAPDH*-R | 5’-ATGGCAACTGTGAGGAG-3’ |
