# Supplementary material for: CXCR2–CXCL1 axis is correlated with neutrophil infiltration and predicts a poor prognosis in hepatocellular carcinoma
Source: J Exp Clin Cancer Res. 2015 Oct 26;34:129. doi: 10.1186/s13046-015-0247-1 (PMC4621872; doi:10.1186/s13046-015-0247-1)
Supplement: Additional file 2: Table S2. — Association of CXCR2 with clinicopathological characteristics. (DOC 66 kb) [file 13046_2015_247_MOESM2_ESM.doc]

**Additional file 2: Table S2.** Association of CXCR2 with clinicopathological characteristics.

|  |  | CXCR2^+^*_IT_* cell | | | CXCR2^+^*_PS_* cell | | | CXCR2^+^*_NT_* cell | | |
| --- | --- | --- | --- | --- | --- | --- | --- | --- | --- | --- |
| Variable |  | Low | High | *P* | Low | High | *P* | Low | High | *P* |
| Age (year) | ≤52 | 71 | 67 | 0.572 | 72 | 66 | 0.496 | 70 | 68 | 0.752 |
|  | >52 | 58 | 63 |  | 58 | 63 |  | 59 | 62 |  |
| Gender | Male | 112 | 114 | 0.834 | 114 | 112 | 0.834 | 111 | 115 | 0.560 |
|  | Female | 17 | 16 |  | 16 | 17 |  | 18 | 15 |  |
| HBsAg | Negtive | 10 | 10 | 0.986 | 8 | 12 | 0.343 | 9 | 11 | 0.654 |
|  | Positive | 119 | 120 |  | 122 | 117 |  | 120 | 119 |  |
| Cirrhosis | Absent | 50 | 46 | 0.574 | 47 | 94 | 0.760 | 54 | 42 | 0.111 |
|  | Present | 79 | 84 |  | 83 | 80 |  | 75 | 88 |  |
| ALT (U/L) | ≤42 | 81 | 73 | 0.277 | 78 | 76 | 0.859 | 81 | 73 | 0.277 |
|  | >42 | 48 | 57 |  | 52 | 53 |  | 48 | 57 |  |
| AST (U/L) | ≤42 | 76 | 74 | 0.745 | 81 | 69 | 0.151 | 78 | 72 | 0.408 |
|  | >42 | 53 | 56 |  | 49 | 60 |  | 51 | 58 |  |
| AFP (ng/mL) | ≤25 | 44 | 45 | 0.932 | 49 | 40 | 0.257 | 42 | 47 | 0.542 |
|  | >25 | 85 | 85 |  | 81 | 89 |  | 87 | 83 |  |
| Tumor size (cm) | ≤5 | 56 | 53 | 0.667 | 59 | 50 | 0.280 | 55 | 54 | 0.858 |
|  | >5 | 73 | 77 |  | 71 | 79 |  | 74 | 76 |  |
| Tumor differentiation | I + II | 56 | 77 | 0.015 | 58 | 75 | 0.040 | 58 | 75 | 0.040 |
|  | III + IV | 71 | 53 |  | 70 | 54 |  | 70 | 54 |  |
| Vascular invasion | Absent | 105 | 105 | 0.898 | 105 | 105 | 0.898 | 105 | 105 | 0.898 |
|  | Present | 24 | 25 |  | 25 | 24 |  | 24 | 25 |  |
| Tumor multiplicity | Solitary | 99 | 95 | 0.496 | 100 | 94 | 0.452 | 101 | 93 | 0.210 |
|  | Multiple | 30 | 35 |  | 30 | 35 |  | 28 | 37 |  |
| TNM stage | I + II | 72 | 67 | 0.490 | 74 | 65 | 0.292 | 74 | 65 | 0.235 |
|  | III + IV | 57 | 63 |  | 56 | 64 |  | 55 | 65 |  |
| BCLC stage | 0-A | 79 | 73 | 0.406 | 79 | 73 | 0.495 | 79 | 73 | 0.406 |
|  | B-C | 50 | 57 |  | 51 | 56 |  | 50 | 57 |  |
| **Note:** Underlined terms indicate statistical significance.  **Abbreviations:** AFP, alpha-fetoprotein; ALT, alanine aminotransferase; AST, aspartate aminotransferase; BCLC, Barcelona Clinic Liver Cancer; CI, confidence interval; HBsAg, hepatitis B surface antigen; TNM, tumor-nodes-metastasis. | | | | | | | | | | |
