# Supplementary figures and images for: CXCR2–CXCL1 axis is correlated with neutrophil infiltration and predicts a poor prognosis in hepatocellular carcinoma
Source: J Exp Clin Cancer Res. 2015 Oct 26;34:129. doi: 10.1186/s13046-015-0247-1 (PMC4621872; doi:10.1186/s13046-015-0247-1)

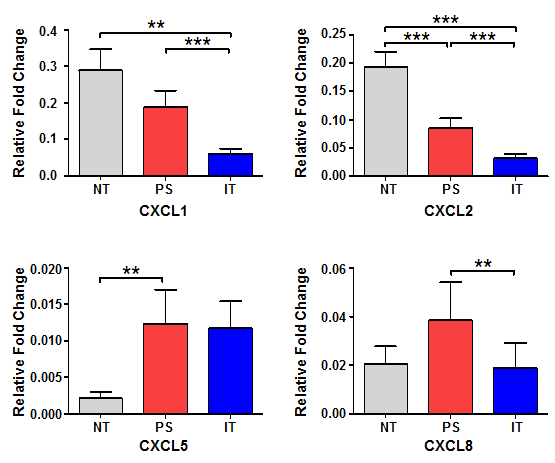

Supplement: Additional file 3: Figure S1. — The gene expression of CXCL1, CXCL2, CXLC5 and CXCL8 in different regions of HCC tissues. Total RNA was extracted from HCC tumor samples and corresponding peri-tumoral and non-tumoral liver tissues. Equal concentrations of total RNA were used in reverse transcription reactions to generate cDNA, then each cDNA was used in SYBR Green real-time quantitative PCR. Data were generated using LightCycler480 software. Levels of target genes were normalized to levels of the expression of a reference gene, GAPDH. Data were processed using GraphPad Prism software; **, P <0.01; ***, P <0.001. (TIFF 45 kb) [file 13046_2015_247_MOESM3_ESM.tif]

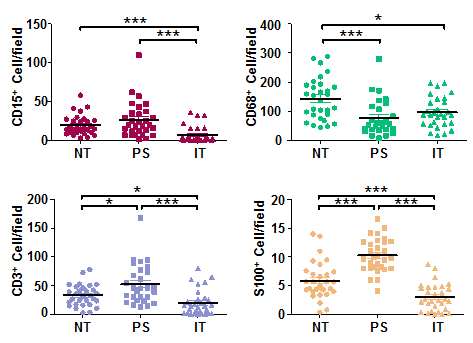

Supplement: Additional file 4: Figure S2. — The number of CD15+, CD68+, CD3+, and S100+ cells in different regions of HCC tissues. Paraffin-embedded HCC sections were stained with anti-CD15, CD68, CD3, or S100 antibodies. The density of CD15+, CD68+, CD3+, and S100+ cells in non-tumoral (NT), peri-tumoral stroma (PS), and intra-tumoral (IT) regions of the same tissue block were calculated (n = 30). Results are expressed as means ± SEM (bars) of groups; *, P <0.05; ***, P <0.001. (TIFF 44 kb) [file 13046_2015_247_MOESM4_ESM.tif]
