# Supplementary material for: CXCR2–CXCL1 axis is correlated with neutrophil infiltration and predicts a poor prognosis in hepatocellular carcinoma
Source: J Exp Clin Cancer Res. 2015 Oct 26;34:129. doi: 10.1186/s13046-015-0247-1 (PMC4621872; doi:10.1186/s13046-015-0247-1)
Supplement: Additional file 5: Table S3. — Univariate and multivariate analyses of factors associated with survival and recurrence. (DOC 40 kb) [file 13046_2015_247_MOESM5_ESM.doc]

**Additional file 5: Table S3.** Univariate and multivariate analyses of factors associated with survival and recurrence.

|  | OS | | | | RFS | | | |
| --- | --- | --- | --- | --- | --- | --- | --- | --- |
|  | Univariate | Multivariate | | | Univariate | Multivariate | | |
| Variables | *P*-value | HR | 95% CI | *P*-value | *P*-value | HR | 95% CI | *P*-value |
| NT CXCL1*CXCR2 (high vs. low) | 0.004 | 0.951 | 0.833–1.085 | 0.456 | 0.007 | 1.032 | 0.927–1.150 | 0.561 |
| PS CXCL1*CXCR2 (high vs. low) | <0.001 | 1.194 | 1.044–1.366 | 0.010 | 0.005 | 1.062 | 0.952–1.185 | 0.284 |
| IT CXCL1*CXCR2 (high vs. low) | 0.038 | 1.063 | 0.920–1.228 | 0.407 | 0.011 | 1.052 | 0.936–1.182 | 0.398 |
